# P18 (SRS35/TgSAG4) Plays a Role in the Invasion and Virulence of *Toxoplasma gondii*

**DOI:** 10.3389/fimmu.2021.643292

**Published:** 2021-06-28

**Authors:** Maguy Hamie, Nadim Tawil, Rana El Hajj, Rania Najm, Sara Moodad, Rita Hleihel, Martin Karam, Sana El Sayyed, Sébastien Besteiro, Marwan El-Sabban, Jean-Francois Dubremetz, Maryse Lebrun, Hiba El Hajj

**Affiliations:** ^1^ Department of Experimental Pathology, Immunology and Microbiology, Faculty of Medicine, American University of Beirut, Beirut, Lebanon; ^2^ Department of Biological Sciences, Beirut Arab University, Beirut, Lebanon; ^3^ LPHI UMR5235, Univ Montpellier, CNRS, Montpellier, France; ^4^ Department of Anatomy, Cell Biology and Physiological Sciences, Faculty of Medicine, American University of Beirut, Beirut, Lebanon

**Keywords:** inflammation, neurotoxoplasmosis, murine toxoplasmosis, cysts, tachyzoites, bradyzoites

## Abstract

Toxoplasmosis is a prevalent parasitic disease caused by *Toxoplasma gondii* (*T. gondii*). Under the control of the host immune system, *T. gondii* persists as latent bradyzoite cysts. Immunosuppression leads to their reactivation, a potentially life-threatening condition. Interferon-gamma (IFN-γ) controls the different stages of toxoplasmosis. Here, we addressed the role of the parasite surface antigen P18, belonging to the Surface-Antigen 1 (SAG-1) Related Sequence (SRS) family, in a cyst-forming strain. Deletion of *P18* gene (KO *P18*) impaired the invasion of parasites in macrophages and IFN-γ-mediated activation of macrophages further reduced the invasion capacity of this KO, as compared to WT strain. Mice infected by KO *P18*, showed a marked decrease in virulence during acute toxoplasmosis. This was consequent to less parasitemia, accompanied by a substantial recruitment of dendritic cells, macrophages and natural killer cells (NK). Furthermore, KO *P18* resulted in a higher number of bradyzoite cysts, and a stronger inflammatory response. A prolonged survival of mice was observed upon immunosuppression of KO *P18* infected BALB/c mice or upon oral infection of Severe Combined Immunodeficiency (SCID) mice, with intact macrophages and natural killer (NK) cells. In stark contrast, oral infection of NSG (NOD/Shi-scid/IL-2Rγnull) mice, defective in macrophages and NK cells, with *KO P18*, was as lethal as that of the control strain showing that the conversion from bradyzoites to tachyzoites is intact and, suggesting a role of P18 in the response to host IFN-γ. Collectively, these data demonstrate a role for P18 surface antigen in the invasion of macrophages and in the virulence of the parasite, during acute and chronic toxoplasmosis.

## Introduction

*Toxoplasma gondii* is an obligate intracellular parasite that infects all warm-blooded animals. Approximately 30% of the population is infected worldwide (1) and the sero-prevalence of *T. gondii* in humans varies between 10 and 70%, according to regions, and significantly increases with age (2–4). Tachyzoites, the rapidly multiplying forms of *T. gondii*, lead to tissue damage and are responsible for acute toxoplasmosis. Tachyzoites use innate immune cells, namely monocytes, macrophages and dendritic cells, to spread into various organs, and subsequently form bradyzoite cysts in the brain and in skeletal muscles (5). These slow-growing bradyzoites are responsible for a persistent disease known as chronic toxoplasmosis (CT). Until recently, parasite persistence in healthy individuals was regarded as clinically asymptomatic. However, an increasing number of reports associate sero-positivity for *T. gondii* with host behavior (6), several neurological disorders (7, 8), and brain cancer incidence (9). In immunocompromised patients, despite the availability of prophylactic and treatment options, reactivation of CT can still occur, imposing a life-threatening situation (10–15). These include Human Immunodeficiency Virus (HIV)-infected patients, cancer patients after chemotherapy, or following bone marrow or organ transplantation (11–15). The interconversion between acute and CT is controlled by the host immune system (16).

Interferon-gamma (IFN-γ) was described as the major mediator of resistance against *T. gondii* (17). IFN-γ-mediated immune response, not only activates macrophages to limit parasite replication, but also provokes intracellular elimination of tachyzoites (17–22). Indeed, during the acute infection, the host’s innate immunity mounts a robust anti-*Toxoplasma* cytokine response, characterized by high interferon-gamma (IFN-γ) production by natural killer (NK) and T cells (21, 23, 24), following IL-12 production by dendritic cells, neutrophils and macrophages (17–20, 22, 25, 26). In the brain, IFN-γ production by brain resident and recruited cells, including microglia and primarily T-cells, is also crucial for the maintenance of cerebral CT latency (27–29). Importantly, IFN-γ regulates the recruitment of T cells into the brain of mice during the acute and chronic phases of the infection (30, 31). In murine CT, CXCL9 and CXCL10 are predominantly expressed in the brains of infected BALB/c mice (32, 33). Furthermore, CXCL9 is crucial to recruit T cells into the brain and to induce their accumulation in zones of tachyzoite proliferation to prevent reactivation of CT (34).

*Toxoplasma* expresses 144 proteins belonging to the SRS family (35). These SRS are differentially expressed in a life cycle stage specific manner, and are potentially involved through mediating attachment to host cells, and regulating the immune response, in the successful initiation of infection. In addition, some SRS proteins play important functions in the context of parasite reactivation. For instance, p36 (SRS9/SRS16B), one of the most abundant bradyzoite-specific proteins plays a major role in both persistence in the brain and reactivation in the intestine (36). Four monoclonal antibodies recognizing four selective pellicular antigens (P36, P34, P21 and P18) were generated against the bradyzoite stage (37, 38). Yet, Expressed Sequence Tag (EST) data reveal the expression of a very low number of transcripts (seven ESTs) of P18 in the tachyzoite stage and a very high number of transcripts (187 ESTs) in the bradyzoite stage, making P18 transcripts amongst the most abundant expressed EST, between the SRS family members (35). The function of P18, encoded by *SAG4/SRS35* (39), remains to be elucidated.

In this study, we investigated the role of P18 during the acute and chronic phases of infection. We showed that P18 deletion doesn’t affect invasion in fibroblasts, while the capacity of invasion of this strain is affected in macrophages. This phenotype of KO *P18* is further affected upon activation of macrophages with IFN-γ. *In vivo*, KO *P18* impacts the virulence of the parasites, by prolonging survival of acute infected mice, in a dose dependent manner. In addition, a lower parasitemia in infected mice, concurrent with a strong elicited immune response manifesting by an increased recruitment of innate immune cells, were observed in mice infected with the KO *P18*. Strikingly, P18 deletion induced a higher number of bradyzoite cysts in the brains of infected mice, along with the induction of immunomodulatory cytokines/chemokines. Furthermore, KO *P18* infected mice exhibited a significantly prolonged survival, upon immunosuppression. Notably, SCID mice, with intact macrophages and NK cells, survived oral infection with the KO *P18* cysts but not with cysts of WT strain. In contrast, NSG mice defective in macrophages and NK cells thus lacking IFN-γ production, succumbed from the infection, suggesting a role of P18 in the response to IFN-γ. Altogether, these results implicate P18 in the invasion of macrophages and in the virulence of the parasite during toxoplasmosis.

## Materials and Methods

### Ethics Statement

All mice protocols were approved by the Institutional Animal Care and Utilization Committee (IACUC) of the American University of Beirut (AUB) (Permit Number: #1312273). All animals were housed in specific pathogen free facility with a 12h ON/OFF light cycle. Humane endpoints were fully respected as per AUB IACUC following AAALAC (Association for Assessment and Accreditation of Laboratory Animal Care International) guidelines and guide of animal care use book (Guide, NRC 2011). Mice were monitored on a daily basis. To verify the acute phase of the infection, blood was withdrawn following deep anesthesia with isoflurane by inhalation. Mice were sacrificed if any abnormal ethical features are noticed as described previously (40). Animals were deeply anesthetized before cervical dislocation.

### Mammalian Cells and Parasite Cultures

Tachyzoites from Pru*Δku80* (WT) [deleted for the *ku80* gene (41)], PruΔ*ku80*Δ*P18* (KO *P18*) or the complemented PruΔ*ku80*Δ*P18+P18* (Cpt *P18*) were used throughout this study. Parasites were maintained by serial passage in human foreskin fibroblasts (HFFs) (American Type Culture Collection-CRL 1634), grown in Dulbecco’s Modified Eagle’s Medium (DMEM) (GIBCO, Invitrogen) supplemented with 10% of fetal bovine serum (FBS), 1% penicillin–streptomycin, 1% kanamycin and 1% glutamine.

The murine macrophage cell line, RAW264.7 (provided by Dr. Aida Habib) was cultured in DMEM medium supplemented with 10% FBS, 1% sodium pyruvate, 1% penicillin–streptomycin, and 1% glutamine (Invitrogen).

Peritoneal macrophages were harvested from BALB/c mice, following their induced recruitment by thioglycollate (38.5 g/L, Sigma). After peritoneal lavage, cells were collected by centrifugation (100 g/10 min). One million murine macrophages were seeded in 6-well-plates and cultured in RPMI medium supplemented with 10% FBS, 1% penicillin–streptomycin, and1% glutamine (Invitrogen). Primary Elicited macrophages were either pre-treated for 24 h with 0.5 ng/ml of IFN-γ or infected for 48 h with WT, KO *P18* and CPT *P18* at the ratio of one parasite for three cells.

### Molecular Cloning

Genomic DNA purification from a pellet of 3 × 108 parasites of WT strain, was performed using wizard Genomic DNA purification system Promega (Ref A230000035819). PCR amplifications were performed on genomic DNA using PrimeSTAR HS DNA/Phusion^®^ High-Fidelity DNA Polymerases (New England Biolabs).

Plasmid pKO-*P18* was designed to replace by double‐homologous recombination, the genomic sequence of *P18* by the open reading frame of *HXGPRT* gene to produce KO *P18* line. A genomic fragment of 2,274 bp corresponding to the 5’ non-coding sequence of *P18* was amplified using ML1514 and ML1515 primers and subcloned into the HindIII and Apa1 sites of plasmid 2854.HX (42). Then a genomic fragment of 2,719 pb corresponding to the 3’ non-coding sequence of *P18* was amplified using ML1516 and ML1517 primers and subcloned at Not I and Spe I sites of plasmid 2854.HX (**Table 1**). Forty micrograms of pKO*P18* was digested by HindIII and Spe1 prior to transfection in the WT strain and was subjected to Mycophenolic Acid and xanthine selection.

**Table 1:** Summary of PCR primers used to generate 5’ *P18*-P2854 HXPRT- 3’ *P18*.

Primers’ names	Primers sequences (5’-3’)			Restriction Enzymes
ML1514	gcgc	GGGCCC	CGATCCGCAGACATCTGGGGGTCTCTTGGCGTTCGTCCCCGCCAACAAAGCG	Apa1
ML1515	ccc	AAGCTT	GGTTGAAGACAGACGAAAGCAGTTGCAGTATGCTGCGACGCGTCTTCCGAG	HindIII
ML1516	gg	ACTAGT	GAGTTCATTGCCAGTGAAGAAGGTGACTGGTAGTGTCACATTTGGCAACTGG	Spe1
ML1517	ataagaat	GCGGCCGC	TTGTTACCTGGCACACGTCACTTGCAACATTGTAAACTTGTTTGTTGTCTGG	Not1

Restriction site specific for each enzyme is underlined and highlighted in yellow and the rest of the primer complementary to the 3’ or 5’ regions is highlighted in green.

To complement the KO *P18* line, we amplified the open reading frame of *P18* flanked by 1,024 pb of 5’ non-coding sequence and 502 bp of 3’UTR and cloned into pLIC-HA vector (43), containing a DHFR selectable marker.

For transfection, 20 × 106 parasites were re-suspended in 800 μl of cytomix (10 mM KPO4, 120 mM KCl, 0.15 mM CaCl2, 5mM MgCl2, 25 mM Hepes, 2 mM EDTA) complemented with 3 mM ATP and 3 mM of reduced glutathione and electroporated with 30–50 μg DNA as described previously (44). Selections of the transgenic parasites were performed with mycophenolic acid (20 μg ml^−1^) and xanthine (50 μg ml^−1^) for HXGPRT selection or pyrimethamine (1 μM) for DHFR-TS selection and chloramphenicol (20 μM) for chloramphenicol acetyltransferase selection. The isolation of clonal transgenic populations was performed using limiting dilution in 96-well plates.

### *In Vitro* Switch From Tachyzoites to Bradyzoites

Confluent HFF cells were cultured in a 6-well plate and on coverslips and infected at a concentration of 1,000 parasites from WT, KO *P18* or Cpt *P18*/well. After a 24-h incubation in complete DMEM medium under 5% CO2, the medium was changed to induction medium (RPMI 1640 without NaHCO3, HEPES 50mM, 3% FBS, pH 8.2). Cells were then maintained in absence of CO2. The basic medium was changed every other day to maintain the pH at 8.2. After 2 weeks, infected cells with bradyzoites were harvested for immuno-fluorescence assay.

### Two-Color Invasion Assays

The Two-color invasion assay was performed as previously described (45). Briefly, five million freshly released tachyzoites from the WT, KO *P18* or Cpt *P18* were added to HFF or RAW 264.7 cells grown on glass coverslips, synchronized on ice for 20 min and subsequently allowed to invade for 5 min in invasion buffer. Invasion was stopped by fixation in 4% paraformaldehyde in PBS and parasites were further processed for IFA. At first, the mouse mAb T_4_ 1E_5_ anti-SAG1 in 2% FBS/PBS was used to detect extracellular parasites. After permeabilization with 0.1% saponin for 15 min, a second IFA was performed using rabbit anti-ROP1 antibodies to label the parasitophorous vacuole of intracellular parasites. Extracellular and intracellular parasites were counted on 40 ± 50 fields per coverslip (three independent experiments).

### Intracellular Parasite Survival Assays

Intracellular parasite survival assays were performed as previously described (40). Only the vacuoles containing two parasites or more were scored. Briefly, HFF or primary macrophage monolayers grown on coverslips in a 24-well plate were infected with 2 × 105 freshly egressed parasites of the WT, KO *P18* or Cpt *P18* strains. After 2 h of incubation at 37°C, the remaining extracellular parasites were removed by five washing in PBS. The infected cells were then left for 24 h at 37°C and fixed with 4% PFA-PBS. Intracellular parasites were stained by IFA using the mouse mAb T_4_ 1E_5_, and the number of total vacuoles was counted.

### Immunofluorescence and Confocal Microscopy

Bradyzoite conversion was confirmed by staining the cyst wall with Biotinylated *Dolichos biflorus* lectin (DBA) (46). Following *in vitro* switch, coverslips of cells infected with cysts of WT, KO *P18* or Cpt *P18* were fixed with 4% paraformaldehyde in PBS for 20 min, permeabilized in Triton (0.2%) for 10 min, blocked for 30 min with 10% FBS in PBS. T_8_3B_1_ or T_8_2C_2_ primary monoclonal antibodies directed against P18 and P34 respectively (37) were used at the dilution of 1:500. Biotinylated DBA (Sigma, Cat. NoB-1035) was used at the dilution of 1:100. Anti-mouse secondary antibody (Abcam, ab150116) were used at the concentration of 1:500. Streptavidin (Sigma) was used at the dilution of 1:100. Coverslips were mounted on slides using a Prolong Anti-fade kit (Invitrogen, P36930). Z-section images were acquired by confocal microscopy using a Zeiss LSM 710 confocal microscope (Zeiss, Oberkochen, Germany) and all images were analyzed using Zeiss Zen software.

### Western Blot Analysis

Following *in vitro* switch, HFF cells infected with cysts of WT, KO *P18* or Cpt *P18* were scrapped, washed with PBS and collected by centrifugation. Pellets were re-suspended in 1× Laemmli buffer and proteins were separated on 10% polyacrylamide gels and transferred to nitrocellulose membranes (BIO RAD Cat# 162-0112). Membranes were probed with the *Dictyostelium* actin recognizing *T. gondii* actin (Gift from Dr. Dominique Soldati-Favre) or with T_8_ 3B_1_ primary monoclonal antibody directed against P18 (37), followed by anti-mouse secondary antibody conjugated to Horseradish peroxidase (HRP) (m-IgGk BP-HRP, Santa Cruz, sc-516102, 1:5,000). GAPDH antibody conjugated to HRP from Abnova (Cat number#MAB5476; 1:20,000) was used as loading control. Bands were visualized using luminol chemi-luminescent substrate (Bio-Rad, Cat# 170-5061).

### Quantitative Real Time PCR

SYBR green qRT PCR was performed using CFX96 (Biorad). SAG-1 primers were used to quantify mRNA representing tachyzoites in wild type WT, KO *P18* or Cpt *P18* strains (**Table 2**). Glyceraldehyde-3-Phosphate dehydrogenase (GAPDH) was used as housekeeping gene (**Table 2**). In qRT-PCR, individual reactions were prepared with 0.25 μM of each primer, 150 ng of cDNA and SYBR Green PCR Master 53 Mix to a final volume of 10 μl. PCR reaction consisted of a DNA denaturation step at 95°C for 3 min, followed by 40 cycles (denaturation at 95°C for 15 s, annealing at the appropriate annealing temperature for each couple of primers (**Table 2**) for 60 s, extension at 72°C for 30 s). For each experiment, reactions were performed in duplicates and the expression of individual genes was normalized to GAPDH Ct values. The Threshold cycle (Ct) corresponds to the cycle at which there is a significant detectable increase in fluorescence. Data were plotted by calculating ΔCt (Cttarget gene − CtGAPDH). Thereafter, ΔΔCt is calculated according to the Livak method: 2^−ΔΔCt^ to obtain the percentage of expression (47).

**Table 2:** Summary of primers used for Real-time quantitative PCR.

Primer	Sequence 5’→3’	Annealing T0C
Mouse GAPDH Forward Primer	5’-CATggCCTTCCgTgTTCCTA-3’	59.4
Mouse GAPDH Reverse Primer	5’-CCTgCTTCACCACCTTCTTgAT-3’	60.3
SAG-1- Forward primer	5’-ACT CAC CCA ACA ggC AAA TC 3’	56.5
SAG-1 Reverse primer	5’-gAg ACT AgC AgA ATC CCC Cg-3’	56.6
Mouse CXCL9 Forward Primer	5’-TgT ggA gTT CgA ggA ACC CT-3’	60.5
Mouse CXCL9 Reverse Primer	5’-TgC CTT ggC Tgg TgC Tg-3’	57.2
Mouse CXCL10 Forward Primer	5’-AgA ACg gTg CgC TgC AC-3’	57.2
Mouse CXCL10 Reverse Primer	5’-CCT ATg gCC CTg ggT CTC A-3’	61.7

### Enzyme-Linked Immunosorbent Assay

Brains from chronically infected BALB/c mice with WT, KO *P18* or Cpt *P18* were harvested at week 4 post-infection (p.i.) with either parasite strain. Following brain homogenization, supernatants were collected, and ELISA was performed using Multi-Analyte ELISArray Kit (Qiagen) according to the manufacturer’s instructions. Briefly, supernatants were spun for 10 min at 1,000*g* and transferred to new Eppendorf tubes, and diluted using a specific cocktail of antigens (IL-12, IL-1β, IFN-γ, IL-6, Tumor necrosis factor-α (TNF-α), IL-10, Monocyte chemoattractant protein 1 (MCP-1), Macrophage Inflammatory Proteins MIP-1α and MIP-1β) provided by the kit (Qiagen). Samples were then transferred to ELISA plate, and were incubated for 2 h. After three washes the detection antibody was added and incubated for 2 h, followed by Avidin-HRP addition for 30 min. Wells were washed and development solution was added in the dark and kept for 15 min, before addition of the stop solution according to manufacturer’s instructions. The optical density (O.D) was determined at 450 and 570 nm and calculated according to the standard values of a positive control according to manufacturer’s instructions.

### *In Vivo* Study

Eight to ten weeks old immunocompetent female BALB/c mice were intra-peritoneally injected with WT, KO *P18* or Cpt *P18* parasites. Mice experimental protocols are indicated in timelines (**Figures 3A–C**, **4B**, **5A**).

To assess the KO *P18* virulence *in vivo*, freshly harvested tachyzoites (10^6^ or 10^5^) of KO *P18* or WT strains were intraperitoneally injected into ten mice per condition (10 mice per condition per experiment, one representative from two independent experiments). Invasiveness of the parasites was evaluated by simultaneous plaque assay of a similar dose of parasites on HFFs. Mouse survival was monitored daily until their death, end-point of all experiments. The immune response of surviving animals was tested day 7 post infection and sera of infected mice were verified by western blotting against tachyzoite lysates.

To assess the pattern of dissemination of the KO *P18* parasites during the acute phase of infection, 1,000 freshly harvested tachyzoites of WT, KO *P18* or Cpt *P18* strains were intraperitoneally injected (Seven mice per condition were tested, one representative from two independent experiments is shown). On day 4 post-infection, mice were sacrificed, peritoneal lavage was performed to screen for tachyzoites number and innate immune cells (macrophages, dendritic cells and natural killer cells) recruitment. Spleens were harvested to assess tachyzoite burden in these organs.

For cyst quantification, iNOS and cytokine production, brains of infected BALB/c mice were harvested at day 28 p.i. To assess the capacity of the KO *P18* to convert from bradyzoites to tachyzoites, ten mice per condition were allowed to establish the chronic infection for 28 days, then mice were immunosuppressed by administrating dexamethasone (Medochemie), at the dose of 5 mg/L in drinking water, and were assessed for survival (five mice per condition per experiment, two independent experiments).

To study the role of IFN-γ, immunocompromised SCID (*Prkdc^scid^*, Charles river) and NSG mice (NOD-*scid IL2Rgamma^null^*, NOD-*scid IL2Rg^null^*, NSG, Jackson laboratory) were orally infected with 20 cysts from WT, KO *P18* or Cpt *P18*, to assess their survival (10 mice per condition per experiment, three independent experiments).

Survival data were represented in Kaplan–Meier plot. Histogram and dot plot analysis were performed using GraphPad Prism 7.

### Immune Cell Identity Staining by Flow Cytometry

BALB/c mice were infected with 1,000 tachyzoites of WT, KO *P18* or CPT *P18* (48) and sacrificed on day 4 post-tachyzoites injection. Peritoneal lavage was performed. Cells recovered from the peritoneum and spleen-derived murine cells from BALB/c mice infected with either parasite line, were stained for 20 min in dark, with anti-CD11b^+^ directly conjugated to Phycoerythrin-Cy7 (PE-Cy7) (BD Bioscience, 2 μg/ml, Clone M1/70), anti-CD11c+ directly conjugated to PE (BD Biosciences, 2 μg/ml, Clone HL3), F4/80 directly conjugated to PE (BD Biosciences, 2 μg/ml, Clone T45-2342) or CD335+ (NKp-46) (BD Biosciences, 2 μg/ml, clone 29A1.4), directly conjugated to Phycoerythrin (PE). Labeled samples were washed twice and 10,000 events were acquired using a FACS ARIAII cell sorter. The flow cytometry results reflect the percentage of different populations of cells in the peritoneum or the spleen (as indicated). Debris and dead cells are gated out by Forward Scatter (FSC) *versus* Side Scatter (SSC) gating.

### Statistics

All *in vivo* experiments were analyzed using Anova one-way test to determine the statistical significance of differences observed between indicated groups for parametric comparisons and presented as averages with standard deviations. Only the secretion levels of cytokines in the brains of infected mice were analyzed using two-tailed Student’s t-tests. Similarly, for invasion assays, we used the ANOVA one way post-hoc Tukey’s and Bonferroni’s multiple comparisons tests to compare the effect of pre-treatment with interferon-γ on each parasite line.

Statistical significance is reported as * for P value between 0.05 and 0.01, ** for P value between 0.01 and 0.001, and ***, or **** for P value less than 0.001.

## Results

### Generation of the *P18* Knock Out and the Complemented Lines

**Figure 1:**
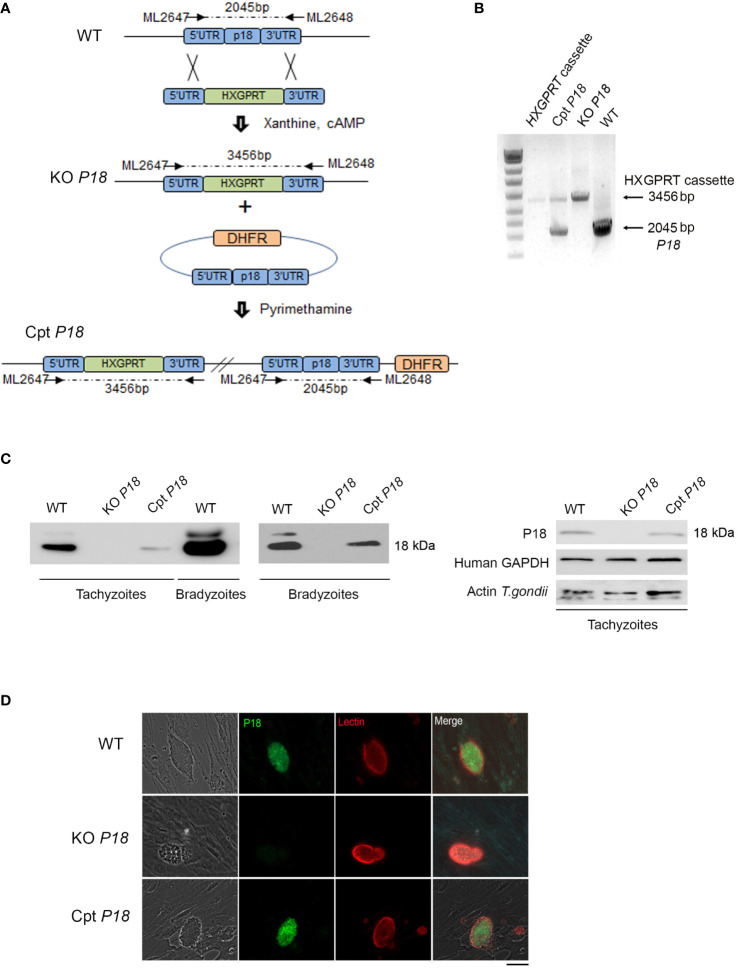
Generation of *P18* knock-out and complemented transgenic lines. **(A)** Schematic representation of the knock-out generation. 5’*P18*-*P2854*-3’*P18* construct is replaced by *P18* by the selectable marker *hypoxanthine–xanthine–guanine phosphoribosyl transferase (HXGPRT)*. 5’*P18*- *P2854*-3’*P18* plasmid was then introduced by electroporation to the WT type II strain to generate KO *P18* parasites. Selection of stably deleted parasites was then performed under Xanthine and Mycophenolic acid. *P18* gene was re-introduced to the KO *P18* for confirmation of its role. The LIC-HA3 vector containing the dihydrofolate reductase (DHFR) selection was used and *P18* was introduced under its own promoter. Following electroporation of the KO *P18* with the LIC-*P18* promoter-*P18*-HA3 generated vector, stable transgenic clones were isolated following pyrimethamine and successful generation of the Cpt *P18* complemented with the *P18* gene was obtained. **(B)** Gel electrophoresis following PCR amplification for the verification of the successful integration of the 5’*P18*-*P2854*-3’*P18* and LIC-*P18* promoter-*P18*-HA3 in the generated KO *P18* and Cpt *P18* transgenic strains respectively. **(C)** Western Blots analysis reflecting the abundance of P18 in the bradyzoites as compared to its low expression in tachyzoites of the WT strain and for the verification of the stable generation of KO *P18* and Cpt *P18* strains, in both tachyzoites and bradyzoites following *in vitro* switch. Human GAPDH and Actin for *T. gondii* were used to ensure the same loading of host and tachyzoites proteins. The results depict one representative experiment among three independent ones. **(D)** Confocal microscopy following IFA assay on *T. gondii* cysts after *in vitro* switch. P18 abrogation is confirmed in the KO *P18* strain (middle panel) as compared to the WT strain (upper panel). *P18* gene is restored in the complemented Cpt *P18* strain (lower panel). P18 protein expression was used using T_8_3B_1_ (green), cysts were stained using a biotinylated lectin (red), with specific binding to a selectin on the cyst wall. Scale bar = 10 μM. The results depict one representative experiment among at least three independent ones.

To gain insights into P18 function, we generated KO *P18* parasites by replacing the corresponding *SRS35/TgSAG4* gene by the selectable marker *hypoxanthine–xanthine–guanine phosphoribosyl transferase (HXGPRT)*, in the WT Pru*Δku80* type II strain (49) (**Figure 1A**). The successful genetic modification was verified by PCR (**Figure 1B**) and the P18 expression level was assessed in tachyzoites and upon *in vitro* switch from tachyzoites to bradyzoites, by immunoblot using specific anti-P18 antibodies (38). Consistent with the published EST data (35), P18 protein levels were abundantly expressed in bradyzoites of the WT strain. Trace levels of P18 were detected in tachyzoites of this strain as they required a higher exposure of the nitrocellulose membrane with Luminol (**Figure 1C**). Importantly, we could confirm P18 abrogation in the KO *P18* strain in both tachyzoites and bradyzoites, upon deletion of its encoding gene (**Figure 1C**). We next generated the Cpt *P18* complemented strain by adding an extra copy of *P18*, under its own promoter (**Figure 1A**). Stable transgenic clone was isolated (**Figure 1B**) and showed expression of P18 by western blot, in both tachyzoites and bradyzoites of Cpt *P18 in vitro* (**Figure 1C**). Consistent with the western blot data, a total extinction of *P18* expression was observed in cysts of the mutant. Differentiating vacuoles was distinguished using fluorescent *Dolichos biflorus* lectin (DBL) which recognizes *N*-acetylgalactosamine on the bradyzoite-specific cyst-wall protein CST1 (50). After 14 days under alkaline stress, the vacuoles of both control and KO *P18* parasites were positive for DBL, but vacuoles of the mutant were negative for P18 stained with anti-P18 antibodies (**Figure 1D**), confirming the loss of expression of P18 in the KO *P18*. P18 expression was restored in the Cpt *P18* strain by IFA (**Figure 1D**). Collectively, these results showed that *P18* deletion and complementation were successful and allow a functional characterization of the P18 protein.

### Deletion of P18 Dramatically Attenuates Parasite Invasion Into Macrophages

**Figure 2:**
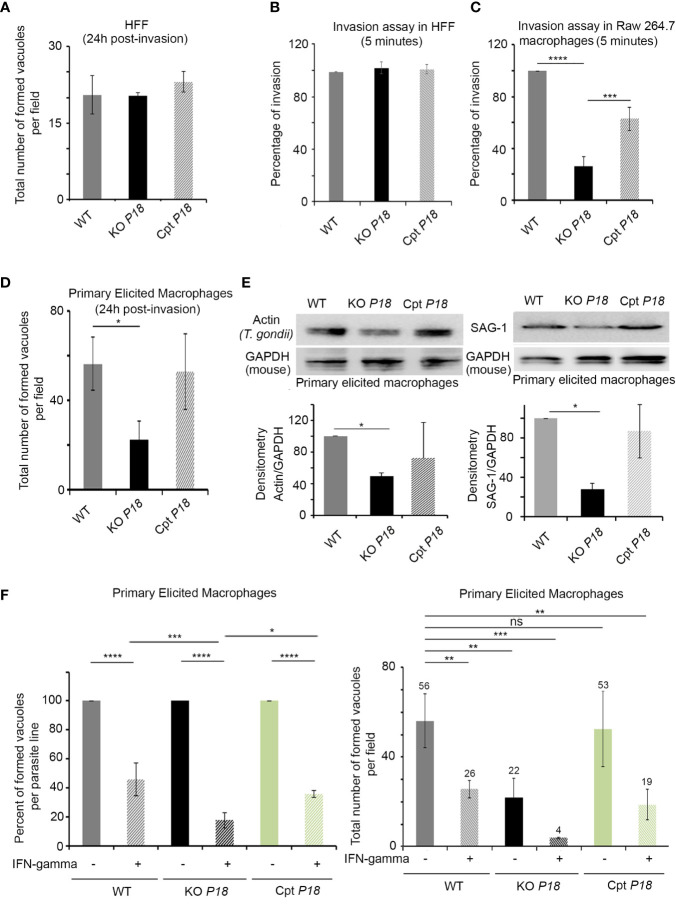
KO *P18* parasites are susceptible to IFN-γ in activated primary macrophages. **(A)** Intracellular parasite survival in Human Forskin Fibroblast (HFF) of WT, KO *P18* or Cpt *P18* assessed by counting the total number of formed vacuoles per field. The result depicts the average for three independent n. The Anova one-way test was performed to validate significance. **(B)** Host cell invasion assay in HFF. WT, KO *P18* or Cpt *P18* parasites were allowed to invade HFF for 5 min before fixation and differential staining of attached extracellular and intracellular parasites. Results were normalized to WT. The result depicts the average for three independent n. The Anova one-way test was performed to validate significance. **(C)** Host cell invasion assay in Raw 264.7. WT, KO *P18* or Cpt *P18* parasites were allowed to invade Raw cells for 5 min before fixation and differential staining of attached extracellular and intracellular parasites. Results were normalized to WT. The result depicts the average for two independent n. The Anova one-way test was performed to validate significance. *, ** and *** indicate P values ≤0.05; 0.01 and 0.001, respectively. P-values less than 0.05 were considered significant. **(D)** Intracellular parasite survival of WT, KO *P18* or Cpt P18 assessed by counting the total number of formed vacuoles per field in primary elicited murine macrophages. The result depicts the average for three independent n. The Anova one way test was performed to validate significance. *, ** and *** indicate P values ≤0.05; 0.01 and 0.001, respectively. P-values less than 0.05 were considered significant. **(E)** Western blot analysis and corresponding densitometry for *Dictyostelium* Actin recognizing *T. gondii* actin (left panel) and SAG-1 (right panel) in the primary elicited macrophages following infection with the WT, KO P18 and CPT P18.The results depict one representative experiment among two independent ones for Actin and three independent ones for SAG-1. The Anova one-way test was performed to validate significance. *, ** and *** indicate P values ≤0.05; 0.01 and 0.001, respectively. P-values less than 0.05 were considered significant. **(F)** Intracellular parasite survival of WT, KO *P18* or Cpt *P18* assessed by counting the percent of formed vacuole for each parasite line in presence of IFN-γ (left panel) and the total number of formed vacuoles per field in primary elicited murine macrophages in absence of IFN-γ and or following their activation with IFN-γ (right panel). The result depicts the average for three independent n. The ANOVA one way post-hoc Tukey’s multiple comparisons test (left panel) and Bonferroni’s multiple comparisons test (right panel) were performed to validate significance. *, **, *** and **** indicate P values ≤0.05; 0.01; 0.001 and 0.0001, respectively. P-values less than 0.05 were considered significant. ns, non significant.

Despite the low expression of P18 in the tachyzoite stage, we investigated the phenotypic consequences of the loss of *P18*. Fresh monolayers of human foreskin fibroblast (HFF) cells were infected with the WT, KO *P18* or Cpt *P18* strains. Deletion of P18 did not affect the number of vacuoles in HFF cells, 24 h post-infection *in vitro* (**Figure 2A**). We then examined the ability of the KO *P18* to invade host cells. We first tested invasion in HFF cells, using a previously established assay to differentiate intracellular from extracellular parasites (45). No remarkable effect on invasion of HFF was observed between the three parasite strains (**Figure 2B**).

Due to the important role of macrophages in determining the outcome of the infection by *T. gondii* (18), we first assessed invasion in this cell type. Monolayers of RAW264.7 murine macrophages were infected with the WT, KO *P18* or Cpt *P18* strains. A significant impaired invasion was demonstrated upon infection with the KO *P18* line (**Figure 2C**). Given that macrophages exhibit significant heterogeneity *in vivo*, we extended our analysis using freshly harvested primary elicited macrophages (PEM). Consistent with the defective capacity of invasion in RAW 264.7, KO *P18* parasites exhibited a lower number of vacuoles, as compared to WT or Cpt *P18*, 24* h* post-infection (**Figure 2D**). This was confirmed using *Dictyostelium* actin recognizing *T. gondii* actin and SAG-1 expression patterns by western blot, which were very similar between Cpt *P18* and WT strains, but lower for KO *P18* in these cells (**Figure 2E**).

In line with several reports [reviewed in (18)], activation of PEM with IFN-γ limited the percentage of vacuoles in the three parasite lines (**Figure 2F**, left panel). Importantly, in IFN-γ activated PEM, the decrease in the total number of vacuoles of the WT parasites was around 50%, while in the KO *P18*, this decrease reached around 85% (**Figure 2F**, right panel). These results suggest a potential role of P18 in the resistance to IFN-γ.

### Deletion of P18 Attenuates Parasite Virulence in Mice

**Figure 3:**
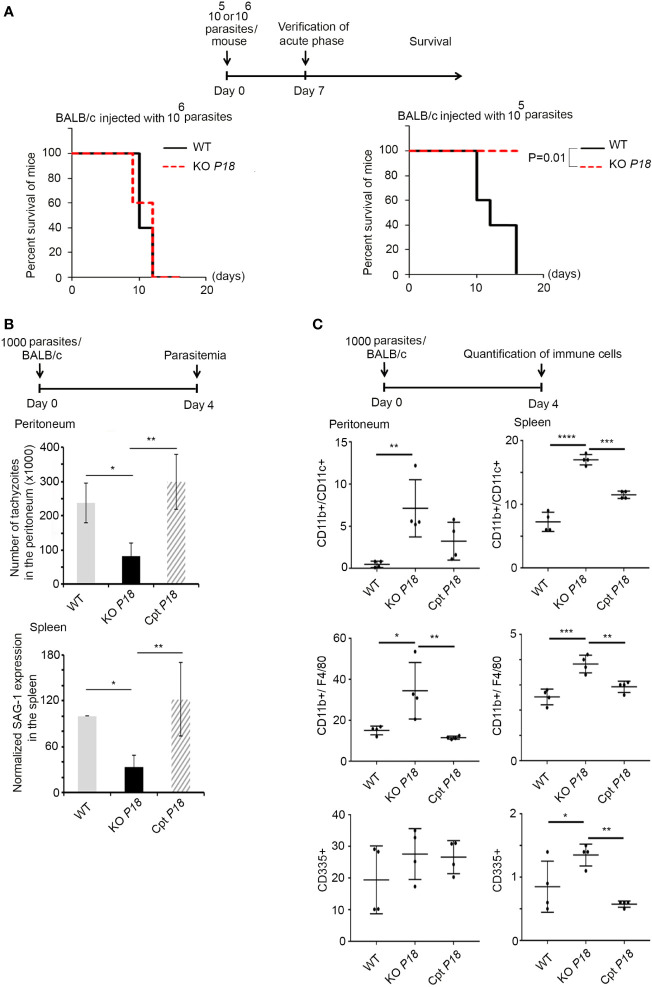
P18 deletion decreases parasite virulence and elicits a strong innate immune response during the acute phase of the infection. **(A)** Timeline schedule for the assessment of lethal parasitic dose. Survival of BALB/c mice infected with 10^6^ (left panel, 10 mice per condition), or 10^5^ (right panel, 10 mice per condition) of WT or KO *P18*. Kaplan–Meier was performed to validate significance (p=0.01 in right panel). *, ** and *** indicate P values ≤0.05; 0.01 and 0.001, respectively. P-values less than 0.05 were considered significant. **(B)** Timeline schedule for assessment of parasitemia during the acute phase of the infection. On day 0, mice were injected with 1,000 tachyzoites/mouse of WT, KO *P18*, or Cpt *P18* strains. Tachyzoites count in the peritoneal lavage (upper panel, five mice per condition), and Quantitative Real-Time PCR for SAG-1 transcripts in spleens of mice injected with WT, KO *P18*, or Cpt *P18* strains and sacrificed at day 4 post-infection (lower panel, three mice per condition). SAG-1 expression was normalized to GAPDH. The results are expressed as percentage of untreated control (±) SD and depict one representative experiment out of two independent ones. The Anova one-way test was performed to validate significance. *, ** and *** indicate P values ≤0.05; 0.01 and 0.001, respectively. P-values less than 0.05 were considered significant. **(C)** Timeline schedule for assessment of immune cells during the acute phase of the infection. On day 0, mice were injected with 1,000 tachyzoites/mouse of WT, KO *P18*, or Cpt *P18* strains. The percentage of CD11b^+^/CD11c^+^ (upper panel, 4 mice per condition), CD11b^+^/F4/80^+^ (middle panel, four mice per condition) and CD335^+^ (lower panel, four mice per condition) were assessed at day 4 post infection as indicated in the peritoneum and spleens of mice injected with WT, KO *P18* and CPT *P18*. Results depict one representative experiment out of two (a total of seven mice per condition, four mice from one n are presented). The Anova one-way test was performed to validate significance. *, **, *** and **** indicate P values ≤0.05; 0.01 and <0.0001, respectively. P-values less than 0.05 were considered significant.

The expression of P18 in the tachyzoite stage, even if low, prompted us to investigate the phenotype of KO *P18* during the acute phase of infection. Inoculation of mice with 10^6^ tachyzoites from the WT or the KO *P18*, resulted in 100% death of mice by 12 days post-infection (**Figure 3A**, left panel). In stark contrast, all mice survived intraperitoneal infection with a lower dose of 10^5^ tachyzoites of the KO *P18* strain, while, mice infected with the WT strain succumbed between 12 and 15 days, reflecting an attenuated virulence upon deletion of *P18 in vivo*.

The difference in the survival phenotype prompted us to further investigate the acute phase of the infection. Mice were thus infected with 1,000 tachyzoites of WT, KO *P18* or Cpt *P18*. Four days post-infection, the number of tachyzoites in the peritoneum of the KO *P18* infected mice was significantly lower than that of the WT infected control mice (**Figure 3B**, upper panel). Similar results were obtained in the spleen, as manifested by the lower transcript levels of SAG-1 in mice infected with the KO *P18*, reflecting less burden of this parasite to this organ (**Figure 3B**, lower panel). This was paralleled with a significant increase in dendritic cells count (around 2-folds) (**Figure 3C**, upper panel), and macrophages (around 2-folds) (**Figure 3C**, middle panel), in both the peritoneum and the spleen of mice infected with KO *P18*, using flow cytometry. A similar significant increase in natural killer cells count was observed in the spleen but not in the peritoneum of mice upon deletion of P18 (**Figure 3C**, lower panel). No effect was observed on the recruitment of T cells at this early time point to the peritoneal cavity (data not shown). To ascertain that these observed *in vivo* phenotypes are due to P18, we tested Cpt *P18* parasites and showed similar tachyzoite numbers to those of WT strain in the peritoneum of infected mice (**Figure 3B**, upper panel). SAG-1 expression patterns were also very similar between Cpt *P18* and WT strains, in the spleens of infected mice with the complemented strain (**Figure 3B**, lower panel). Altogether, these results show that the observed phenotypes implicate P18 in the virulence of the parasite. The higher recruitment of innate immune cells upon infection with the KO *P18*, may explain this attenuated virulence during the acute phase of the infection.

### P18 Deletion Increases the Number of Bradyzoite Cysts *In Vitro* and *In Vivo*

**Figure 4:**
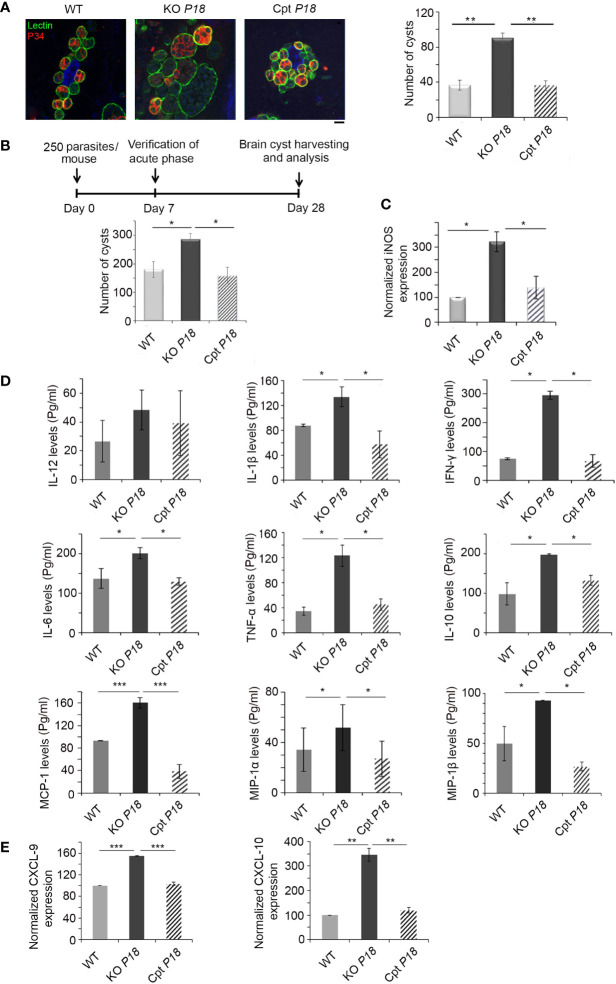
KO *P18* parasites form more bradyzoite cysts *in vitro* and *in vivo*. **(A)** Quantification and size of cysts by confocal microscopy following IF assay, after *in vitro* switch using a biotinylated lectin (green), with specific binding to a selectin on the cyst wall. The results depict one representative experiment among three independent ones. Number of cysts was determined in 50 independent fields per condition. Scale bar = 2 μM. **(B)** Timeline schedule for assessment of bradyzoite formation/number following *P18* deletion in BALB/c mice. Briefly, on day 0, BALB/c mice were injected with 250 tachyzoites/mouse of the WT, KO *P18*, or Cpt *P18* strains. Acute toxoplasmosis was verified on day 7 p.i. On day 28 p.i., Brains of *Toxoplasma* positive mice were harvested. Cyst count following Percoll extraction (10 mice per condition). The results are expressed as percentage of untreated control (±) SD and depict one representative experiment among two independent ones. The Anova one-way test was performed to validate significance. *, ** and *** indicate P values ≤0.05; 0.01 and 0.001, respectively. P-values less than 0.05 were considered significant. **(C)** Quantitative Real-Time PCR for iNOS (right panel, 10 mice per condition) from brains of mice injected with WT, KO *P18*, or Cpt *P18* strains. iNOS expression was normalized to GAPDH. **(D)** ELISA showing the secretion levels of different cytokines/chemokines (IL-12, IL-1β, IFN-γ, IL-6, TNF-α, IL-10, MCP-1, MIP-1α and MIP-1β) in BALB/c mice chronically infected with the WT, KO *P18*, or Cpt *P18* strains (five mice per condition). The t-test was performed to validate significance. *, ** and *** indicate P values ≤0.05; 0.01 and 0.001, respectively. P-values less than 0.05 were considered significant. The results depict one representative out of two independent experiments. **(E)** Quantitative Real-Time PCR for CXCL-9 and CXCL-10 (five mice per condition) from brains of mice injected with WT, KO *P18*, or Cpt *P18* strains. CXCL-9 and CXCL-10 expressions were normalized to GAPDH. The Anova one-way test was performed to validate significance. *, ** and *** indicate p values ≤0.05; 0.01 and 0.001, respectively. P-values less than 0.05 were considered significant. The results are expressed as percentage of untreated control (±) SD and depict one representative experiment among two independent ones for **(B–E)** panels.

It is well documented that P18 is expressed in the bradyzoite stage, at both the transcript and protein levels (35, 39). We assessed the effect of P18 deletion on bradyzoite cyst formation and number by quantifying lectin-positive cysts, in several fields by immunofluorescence microscopy. After 14 days of *in vitro* conversion from tachyzoites of WT, KO *P18* or Cpt *P18* strains, increased number of cysts was observed in the P18 deleted strain, suggesting a higher number of bradyzoites (**Figure 4A**).

We then investigated the number of brain cysts *in vivo* using intraperitoneal injection of mice with a non-lethal dose of 250 parasites of WT, KO *P18* or Cpt *P18* strains. The acute phase was verified seven days post infection by immune reactivity of infected mice on tachyzoite extracts (51). Twenty-eight days post infection, brains of infected mice with the three different strains were harvested for cyst quantification (Timeline described in **Figure 4B**). P18 deletion significantly increased the number of cysts in the brains of infected mice (**Figure 4B**), confirming the *in vitro* finding. Complementation of *P18* in the Cpt *P18* strain reverted the observed phenotype (**Figure 4B**). Altogether, these results demonstrate that P18 impacts bradyzoite cyst number *in vitro* and *in vivo*.

### P18-Deficient Bradyzoites Elicit a Stronger Brain Immune Response

Nitric-oxide (NO), produced by microglial cells and macrophages that infiltrate the CNS, inhibits *T. gondii* replication and plays a vital role in the progression of the infection (52–57). Inducible nitric oxide synthase (iNOS) expression is also up-regulated in the brains of mice infected with type II strains (58). Consistent with the higher number of cysts obtained upon *P18* deletion (**Figure 4B**), iNOS transcriptional levels were elevated in the knock out strain (**Figure 4C**). This increase of expression was restored to WT level, in the complemented strain, suggesting a potential role for NO *via* iNOS up-regulation in mediating stress responses, that might lead to a higher conversion of tachyzoites of the KO *P18* into bradyzoite cysts.

IFN-γ response is also crucial upon establishment of CT and in the control of cerebral *T. gondii* growth (28). IFN-γ elicits intracerebral immune response by the production of cytokines and chemokines (5, 16, 17, 25, 28, 33). We showed that, upon deletion of P18, the secreted levels of IL-1β, IFN-γ, IL-6, TNF-α, IL-10, MCP-1, MIP-1α and MIP-1β were specifically up-regulated in the brains of chronically infected mice (**Figure 4D**). IL-12 secreted levels were marginally upregulated but not statistically significant (**Figure 4D**). Complementation with P18 restored cytokine/chemokine levels to those induced by the WT strain (**Figure 4D**). These results indicate that the KO *P18* strain induces a stronger immune response in the brains of infected mice.

During murine CT in BALB/c mice, CXCL9 and CXCL10, are predominantly expressed in the brains of infected mice (32, 33). Furthermore, CXCL9 is crucial to recruit T cells into the brain and to control reactivation of CT (34). Interestingly, P18 deletion led to a significant upregulation of both CXCL9 and 10, suggesting the recruitment of T cells to mount the needed IFN-γ immune response to control the infection (**Figure 4E**).

### Immunosuppressed BALB/c and SCID Mice Exhibit a Prolonged Survival Upon Infection With KO *P18* Parasites

**Figure 5:**
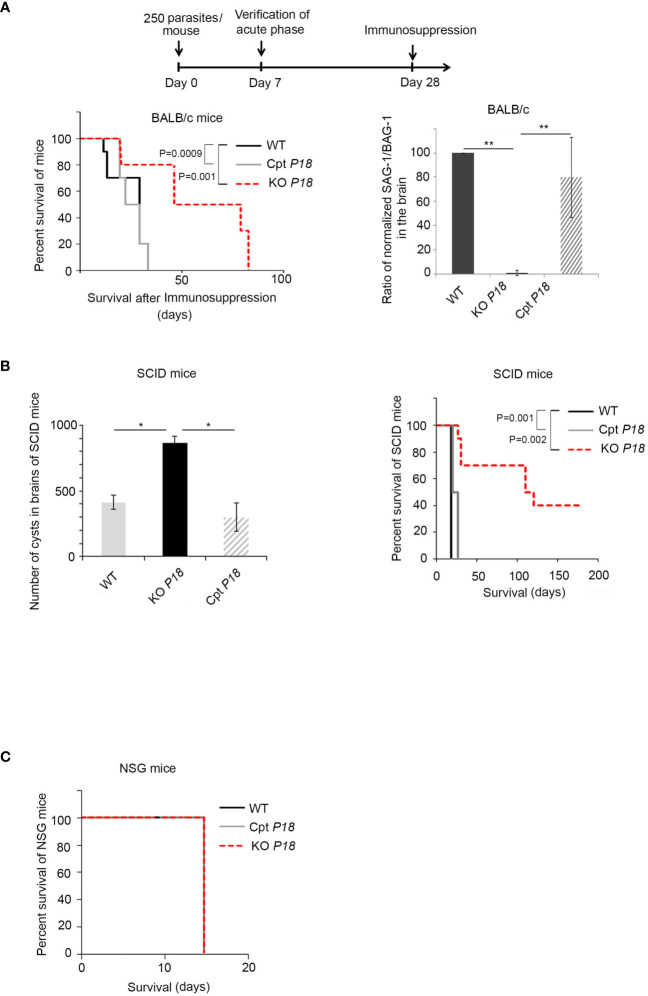
KO *P18* infected mice are susceptible to IFN-γ response upon immunosuppression **(A)** Timeline schedule for establishing chronic infection in BALB/c mice and stimulating immunosuppression. On day 0, mice were injected with 250 tachyzoites/mouse of WT, KO *P18*, or Cpt *P18* strains. Acute toxoplasmosis was verified on day 7 post-infection. On day 28, mice were treated with the immunosuppressive dexamethasone drug until death. The survival of immunosuppressed BALB/c mice infected with WT, KO *P18*, or Cpt *P18* strains following reactivation of chronic toxoplasmosis (left panel, 10 mice per condition, three independent experiments). Kaplan–Meier was performed to validate significance (p = 0.001 for WT vs KO and p = 0.0009 for CPT P18 vs KO P18). *, ** and *** indicate P values ≤0.05; 0.01 and 0.001, respectively. P-values less than 0.05 were considered significant. Ratio of normalized transcript levels SAG-1/BAG-1 from mice injected with WT, KO *P18*, or Cpt *P18* strains when they start to die after immunosuppression (day 30 after dexamethasone, right panel, five mice per condition, one representative out of three independent experiments). In individual mice SAG-1 and BAG-1 expression were normalized to GAPDH, before showing the ratio. The results are expressed as percentage of untreated control (±) SD. The Anova one-way test was performed to validate significance. *, ** and *** indicate P values ≤0.05; 0.01 and 0.001, respectively. P-values less than 0.05 were considered significant. **(B)** Number of cysts in brains of SCID mice following oral gavage with 20 cysts from WT, KO *P18* or Cpt *P18* (left panel, 10 mice per condition, one representative out of two independent experiments) and Survival of SCID following oral infection with cysts (as indicated) (right panel, 10 mice per condition, one representative out of two independent experiments). Kaplan–Meier was performed to validate significance. * and ** indicate P values ≤0.05; 0.01 and 0.001, respectively. P-values less than 0.05 were considered significant. **(C)** Survival of NSG mice (10 mice per condition one representative out of two independent experiments) following oral gavage with 20 cysts of the WT, KO *P18*, or Cpt *P18* strains.

The higher number of brain cysts prompted us to address the potential role of P18 following immunosuppression. We used continuous dexamethasone administration in drinking water starting at day 28, time point at which CT is established in the brains of infected mice CT (59) (Timeline described in **Figure 5A**). Immunosuppression of mice infected with the WT parasites led to 100% lethality between 29 and 33 days post-dexamethasone administration (**Figure 5A**, left panel). Interestingly, survival of mice infected with KO *P18* parasites was significantly extended after immunosuppression. Death was recorded between days 80 and 81 after initial administration of dexamethasone (**Figure 5A**, left panel).

Upon complementation with *P18*, the survival phenotype was reverted, and animals succumbed between days 28 and 33 post-dexamethasone administration (**Figure 5A**, left panel), indicating that the observed phenotype is due to *P18* deletion. We then examined the ratio tachyzoites to bradyzoites following immunosuppression. We quantified the ratio of transcript levels of the tachyzoite marker SAG-1/the bradyzoite marker BAG-1 in the brains of infected mice. Since the average time of death of mice infected with control or complemented strains was day 30 post-dexamethasone administration, we assessed SAG-1/BAG-1 expression at this specific time point. Consistent with the survival results, the ratio SAG-1/BAG-1 was significantly lower in the brains of infected mice with the KO *P18* parasites (**Figure 5A**, right panel). This result may reflect either a deficit in the conversion of bradyzoites to tachyzoites, or an intact conversion, yet a clearance of tachyzoites by the immune system especially that KO *P18* tachyzoites have a defective invasion of macrophages and are more susceptible to IFN-γ response.

To explain the conferred survival advantage in mice infected with the KO *P18*, we took advantage of the difference between SCID and NSG mice. SCID mice lack adaptive B and T cells related immunity, but yet retain an innate immunity with intact macrophages, antigen-presenting cells, and NK cells (60). In contrast, NSG model is defective for macrophage and NK activity and presents deletion or truncation of the gamma chain of interleukin 2 (IL-2) receptor (61). Some 20 cysts harvested from the brains of BALB/c mice infected with WT, KO *P18* or Cpt *P18* were administered by oral gavage to SCID or NSG mice. The lack of adaptive immune response in these mice led us to verify the successful infection by assessing the capacity of SCID mice to develop brain cysts, a result that implies an initial conversion into tachyzoites, dissemination of tachyzoites and then conversion to bradyzoites. Consistent with our results (**Figure 4B**), the highest number of cysts was obtained upon oral infection of SCID mice with the KO *P18* cysts (**Figure 5B**, left panel). Interestingly, while SCID mice infected with the WT or Cpt *P18* succumbed between 15 and 18 days post-oral infection, SCIDs infected with KO *P18* parasites, showed a significant prolonged survival (26–122 days) in 60% of infected animals, while the remaining 40% of mice were still alive after an extended period exceeding more than 180 days (**Figure 5B**, right panel). Conversely, oral gavage of NSG mice with 20 cysts resulted in 100% death of mice after 15 days post-infection (**Figure 5C**), which reflects a first step of conversion of bradyzoite to tachyzoite and eventually active proliferation of tachyzoites during lethal acute phase. Altogether, these results exclude the possibility of a defective conversion from bradyzoites to tachyzoites in KO *P18*. Since KO *P18* killed immunodeficient mice defective in macrophages and NK, but not immunodeficient mice that retained the activity of these cells, this suggests that P18 might be involved in a step that plays a role in the ability of the parasite to grow in a permissive IFN-γ environment, or to respond to NK-mediated cytotoxicity, hence explaining the prolonged survival in SCID but not in NSG mice.

## Discussion

Our study demonstrates a role for P18 in invasion of macrophages and also implicates this protein in the virulence of the parasite. A critical step in *T. gondii* pathology is the interconversion between tachyzoite and bradyzoite stages, that determine persistence and transmission of the parasite. The superfamily of SRS genes comprises 144 members, of which 35 are pseudo genes (35). Differential expression of SRS during life cycle stages of the parasite is essential for the initiation of infection, modulation of host immunity and establishment of transmissible infections (62–64). Of all the recognized proteins, the tachyzoite-specific SAG1/SRS29B functions as an adhesin, influences virulence and induces lethal ileitis in mice (41, 63, 65–68). In addition, SAG1/SRS29B, along with other tachyzoite-specific members like SRS34A, and SRS29C proteins, elicit high antibody titers during the acute phase of the infection (69). We highlighted the role of another member of this family, SAG4/SRS35 (P18) in the invasion of macrophages, and demonstrated a stronger immune response in absence of this protein during the acute and chronic phases of the infection. Although P18 is abundantly expressed in bradyzoites, it still exhibits a phenotype during the acute phase of the infection. One can speculate that P18 (SAG-4) may have an effect on P30 (SAG-1) expression, which is specific to tachyzoites. However, when we tested the expression of SAG1 in KO *P18* strain, no difference of expression was noted as compared to WT parasites (data not shown), making this hypothesis less likely. While SAG1 is well known to elicit a strong immune response during the acute infection, P18 seems to lower this response. P18, even if dismally expressed in tachyzoite, may counter balance this high immune response, hence escaping host elimination, but this hypothesis requires future in-depth investigation.

Immune response is essential to drive the switch to latent bradyzoites expressing specific members of this family. Upon establishment of CT, other members of this family play pivotal functions. For instance, CST1/SRS44, a mucin-like domain glycoprotein, is essential to construct and maintain an intact and rigid cyst wall (50, 70). Another protein, SRS13 was identified and localized to the cyst wall and matrix. However, unlike CST1/SRS44, SRS13 is not necessary for the assembly of the cyst wall (71). Two other cyst wall proteins, CST2 and CST3, were also studied and their respective knock out strains revealed a normal phenotype with respect to growth or cyst formation *in vitro*, yet, CST2-KO parasites were markedly less virulent during the acute infection in mice (44). SRS9/SRS16B encodes an abundant bradyzoite-specific protein, p36 (36). SRS9 plays an important role in both persistence in the brain and reactivation in the intestine (36).

In our study, we identified a role for P18 in the invasion of macrophages, and virulence during the acute phase of the infection. More importantly, we identified a role of this protein during cyst formation. Unexpectedly, P18 seemed to be involved in controlling cyst size during *in vitro* differentiation in HFFs but this observation requires further investigation. In contrast to the reported function of p36, P18-deleted parasites resulted in more cysts *in vitro* and *in vivo*. Despite this higher number of cysts, a prolonged survival of mice was obtained upon dexamethasone-induced immunosuppression only in mice having intact macrophage and NK activity. The observed phenotypes, are unlikely to reflect a defective role in interconversion between bradyzoites and tachyzoites in the KO *P18* for the following two reasons. First, after oral gavage of SCID mice, the *P18* mutant was able to form cysts into the brain of infected mice, a result that implies their initial conversion into tachyzoites, dissemination of tachyzoites and then conversion to bradyzoites. Second, in NSG mice, infection with all parasite lines resulted in 100% death at the same time. This implies also that P18 bradyzoites were able to convert into tachyzoites before they disseminate as tachyzoites and kill mice. The survival observed in SCID mice may be either attributed to the defective invasion of macrophages, which play a role in the dissemination of the parasites, or also to the higher susceptibility of the KO *P18* to IFN-γ response, presumably produced by NK cells in these mice. A third possibility entailing an NK-mediated cytotoxicity, which could be controlling the KO *P18* strain in SCID mice independently of IFN-γ is also plausible and requires further investigation. Treatment of SCID mice with an anti-NK antibody to neutralize its function may clarify this hypothesis.

IFN-γ, pivotal in both innate and adaptive immune responses to infection with *T. gondii*, plays a role in the activation of macrophages to limit the parasite replication [For a review (18)]. We demonstrated that upon activation of PEM with IFN-γ, KO *P18* is more susceptible than the WT parasite, suggesting a potential role of P18 in the resistance to this cytokine, hence contributing to the virulence of the parasites. This was also supported by the dose-dependent virulence obtained during the acute phase of infection, upon deletion of P18. Indeed, several studies reported that *T. gondii* evolved mechanisms to subvert macrophage activation, and to use their migratory activities to promote dissemination [For a review (18)]. Our results may position P18 as one of these parasite adhesins, to increase invasion in macrophages, presumably to resist to IFN-γ induced immune response, to promote the fast potential dissemination of the parasite, contributing to its persistence. It was previously reported that the parasite SRS protein SAG2A induces phenotypic and classical activation of macrophages in mice during the acute phase of the disease (72), suggesting that different members of the SRS family play different function on host immunity. While some induce the activation of the innate immune response, other members seem to resist to this response. Activation of macrophages induces inhibition of tachyzoite multiplication *via* iNOS, arginase-1 as well as the expression of the inhibitory proteins indoleamine 2,3-dioxygenase (IDO) and the expression of the effector proteins immunity-related GTPases (IRGs) (73). Furthermore, it is documented that NK cells, CD8 T cells and CD4 T cells mediate cytotoxicity and high amounts of IFN-γ (74), a dominant factor that enhances the ability of macrophages to destroy *T. gondii* (73). Infection of mice with the P18-deleted strain elicited a higher recruitment of macrophages, dendritic cells and NK cells to the peritoneal cavity. This implicates P18 in the resistance to the inflammatory process to escape the pressure of macrophages during the early onset of the infection. Whether P18 acts at the level of inflammasome, or other enzymes implicated in the macrophage toxoplasmicidal effect is yet to be elucidated.

Dendritic cells represent a major forefront exploited by the parasite, due to their capacity to secrete defense molecules, present antigens mediating crosstalk to T cells, but most importantly due to their shuttling role to various organs. Indeed, infection with *T. gondii* induces increased numbers of DCs at the site of infection, then in the spleen, and lymph nodes (24). We demonstrated that KO *P18* elicited a higher number of DCs to the peritoneum and the spleen of infected mice. Infection with the cyst-forming strains of *T. gondii* leads to both higher migration and hypermotility of DCs (75–79), helping in the dissemination of these strains to various tissues, escaping the host inflammatory response (79). The highest number of recruited DCs, along with an increased cyst number in the brains of infected mice with KO *P18*, may suggest a higher migration of infected DCs to reach the brain and evade the immune response. This possibility requires further investigation.

Brain-resident cells contribute to the intracerebral immune response by the production of cytokines, chemokines and expression of immune-regulatory cell surface molecules (80). Wandering immune cells are also recruited to the site of infection in the CNS and contribute to the response against the infection (80). In line with these studies, we demonstrated a higher brain host immune response explaining the higher number of cysts upon P18 deletion.

IFN-γ plays a major role during CT establishment and persistence. Notably, brain recruited DCs are the main producers of IL-12, which is crucial for the maintenance of IFN-γ during the latent phase (81). NK can produce IFN-γ but the main source of this cytokine remains the recruited T cells, which infiltrate into the brain following infection (31, 80). IFN-γ can be also produced by microglia, leading to their activation and the production of NO, which controls CT (29, 31, 80, 82). The elevated levels of iNOS in the brains of infected mice with the KO *P18* strain, may reflect an increase of NO, to control CT. During CT, CXCL9 and CXCL10, among others, play important roles in recruiting T cells and macrophages into the brain to maintain the latency of infection (83). In line with the higher number of cysts, CXCL9 and CXCL10 were significantly upregulated in the brains of mice infected with the KO *P18* strain, presumably playing a role in the maintenance of CT (27–29). Our results showing a prolonged survival obtained in SCID mice where IFN-γ production by NK cells is not impaired, suggests, not only a role of T cells in producing this cytokine for maintenance and persistence of the bradyzoite stage, and the control of recrudescence to tachyzoites (27), but also involves innate immune cells in its production. The susceptibility of KO P18 tachyzoites to IFN-γ and the defect of invasion of macrophages which might reduce the dissemination in both peritoneum and spleen during the acute phase of the infection, may also explain the prolonged survival in immunosuppressed SCID and BALB/c mice. Indeed, a plausible scenario would be less tachyzoite dissemination and/or more tachyzoite killing in the presence of IFN-γ. To confirm the direct role of P18 on IFN-γ, the susceptibility of KO *P18* in IFN-γ null mice or treatment of SCID mice with anti-IFNγ neutralizing antibodies remain necessary.

Overall, our results identify a role for another member of the SRS family in the invasion of macrophages, and virulence of the parasite, at multiple levels within the intermediate host. This contributes to the general understanding of toxoplasmosis, a disease that poses significant health and economical burdens, with implications in both immunocompetent and immunocompromised patients where recurrence of CT is severely morbid and potentially lethal, especially that the recent recommendations consider toxoplasmosis as a neglected parasitic infection, requiring public health action (CDC 2019).

## Data Availability Statement

The original contributions presented in the study are included in the article/supplementary material. Further inquiries can be directed to the corresponding author.

## Ethics Statement

The animal study was reviewed and approved by the Institutional Animal Care and Utilization Committee (IACUC) of the American University of Beirut (AUB) (Permit Number: #1312273).

## Author Contributions

MH, NT, REH, RN, SM, RH, MK, and SS performed experiments. HH, SB, ML, J-FD, and ME-S analyzed results. MH, ME-S, and HH made the figures. HH designed the research. HH and MS wrote the paper. All authors contributed to the article and approved the submitted version.

## Funding

This work was made possible through core support from the Medical Practice Plan (Faculty of Medicine, American University of Beirut) and the American University of Beirut and the Centre National de Recherche Scientifique Libanais (AUB‐CNRS‐L GRP) funds.

## Conflict of Interest

The authors declare that the research was conducted in the absence of any commercial or financial relationships that could be construed as a potential conflict of interest.
